# Population expansion and individual age affect endoparasite richness and diversity in a recolonising large carnivore population

**DOI:** 10.1038/srep41730

**Published:** 2017-01-27

**Authors:** Ines Lesniak, Ilja Heckmann, Emanuel Heitlinger, Claudia A. Szentiks, Carsten Nowak, Verena Harms, Anne Jarausch, Ilka Reinhardt, Gesa Kluth, Heribert Hofer, Oliver Krone

**Affiliations:** 1 Leibniz Institute for Zoo and Wildlife Research, Alfred-Kowalke-Straße 17, 10315 Berlin, Germany; 2 Humboldt-Universität zu Berlin, Ecology and Evolution of Molecular Parasite Host Interactions, Philippstraße 13, 10115 Berlin, Germany; 3 Senckenberg Research Institute and Natural History Museum Frankfurt, Clamecystrasse 12, 63571 Gelnhausen, Germany; 4 LUPUS Institute for Wolf Monitoring and Research in Germany, Dorfstraße 20, 02979 Spreewitz, Germany

## Abstract

The recent recolonisation of the Central European lowland (CEL) by the grey wolf (*Canis lupus*) provides an excellent opportunity to study the effect of founder events on endoparasite diversity. Which role do prey and predator populations play in the re-establishment of endoparasite life cycles? Which intrinsic and extrinsic factors control individual endoparasite diversity in an expanding host population? In 53 individually known CEL wolves sampled in Germany, we revealed a community of four cestode, eight nematode, one trematode and 12 potential *Sarcocystis* species through molecular genetic techniques. Infections with zoonotic *Echinococcus multilocularis, Trichinella britovi* and *T. spiralis* occurred as single cases. Per capita endoparasite species richness and diversity significantly increased with population size and changed with age, whereas sex, microsatellite heterozygosity, and geographic origin had no effect. Tapeworm abundance (*Taenia* spp.) was significantly higher in immigrants than natives. Metacestode prevalence was slightly higher in ungulates from wolf territories than from control areas elsewhere. Even though alternative canid definitive hosts might also play a role within the investigated parasite life cycles, our findings indicate that (1) immigrated wolves increase parasite diversity in German packs, and (2) prevalence of wolf-associated parasites had declined during wolf absence and has now risen during recolonisation.

Biodiversity describes the variety of organisms sharing an ecosystem and can be measured in levels of genetic variation, the number of occurring species (species richness) or by determining species diversity when accounting for the number of species and their abundance^1^. The respective ecosystems can be of different dimension, ranging from a single individual serving as host ecosystem for a community of microorganisms, to a local population in a distinct environment up to a global scale. In conservation biology, measuring biodiversity is a crucial tool to assess the (health) state of the ecosystem of interest^2^.

The factors responsible for the presence and diversity of parasites in free-ranging mammalian host populations have been the subject of an increasing number of investigations in the past two decades^3^,^4^,^5^,^6^,^7^,^8^,^9^. These include external factors such as host population density and geographical location, and intrinsic factors such as genetic constitution, life history, and other conditions which may vary between individuals and host populations. Most of these studies have been conducted on rodents^7^,^8^,^9^. Many have investigated the drivers of parasite diversity across several species, while only few intraspecific studies have considered carnivores as hosts^10^,^11^, particularly ecologically important apex predators^12^, and even fewer have either been experimental in nature or used natural events that correspond to a quasi-experimental study design^13^,^14^. The typical framework of these studies has been a reasonably stable ecological setting within which the host population(s) under scrutiny has existed at the study site within living memory. The consequences for parasite presence and diversity are thus not clear, should a host population, particularly an apex predator, go extinct and recolonise its habitat almost a century later. Such extinction events correspond to a quasi-experimental set-up. It allows addressing questions such as: How would parasite diversity be affected by a small host founder population; to what extent do extrinsic and intrinsic factors control parasite diversity for individual hosts in an expanding host population; and which role do prey populations play in the re-establishment of parasite life cycles and parasite transmission for predator hosts? Here we use a recent and intriguing case of a recolonising and expanding apex predator, the grey wolf (*C. lupus*) in Central Europe to study these questions.

After having been eradicated for almost a century from Central Europe, grey wolves returned to Germany during the late 1990s and established the first breeding pack in the year 2000^15^. The first individuals immigrated from the Baltic wolf population from North Eastern Poland^15^,^16^. Since then, the population has rapidly expanded, leading to the establishment of the current Central European lowland (CEL) wolf population across Northern Germany and Western Poland. As of 2015, at least 39 breeding packs and pairs live in Germany, and at least 30 packs and pairs occupy Western Poland^17^. This newly established CEL wolf population provides an opportunity to study some additional and – in the context of conservation management – highly relevant questions on host-parasite relationships. In contrast to study sites in North America or Africa with a minor overlap between predators and people, people and wildlife in Central Europe coexist in an anthropogenically modified cultural landscape with a high human population density^18^. Here, transmission of pathogens between wolves, companion animals, livestock and people may easily occur^19^ because free-ranging grey wolf populations are hosts of and vectors for a variety of macro^20^- and microparasites^21^ which circulate in sylvatic and domestic cycles. Both pathogen spillover and spillback may occur and affect wild and domestic species, threaten human health^19^, and in the case of livestock may even have an economic impact^22^.

Such issues are especially accessible to investigation in eukaryotic parasites, establishing more stable host-parasite interactions compared to bacteria and viruses. Amongst helminths, the larval stages of taeniid species are known to cause health problems in people and livestock. They require a two-host cycle, with an intermediate host developing the metacestode/cysticercus and a predator definitive host consuming it and developing the mature tapeworm. Local diet analyses of wolves have demonstrated that roe deer (*Capreolus capreolus*), red deer (*Cervus elaphus*), and wild boar (*Sus scrofa*) are the main prey species of the newly expanding wolf population in Germany^23^, and may therefore serve as intermediate hosts of typical wolf endoparasites. For instance, the cestode *Taenia krabbei* might play a crucial role as intestinal parasite in European wolves^24^ and is known to develop metacestodes in cardiac and skeletal muscles in intermediate hosts^24^,^25^. In contrast to *T. krabbei*, for which no human case of cysticercosis has been reported so far, other tapeworm species have a high zoonotic potential^26^ and are responsible for several types of cysticercoses (*T. hydatigena*)^27^, coenuroses (*T. multiceps*)^28^, and echinococcoses (*E. multilocularis, E. granulosus*)^29^. In addition, nematodes and trematodes spread by carnivores are known to cause trichinellosis (*Trichinella* spp.)^30^ and alariosis (*Alaria alata*)^31^ in people, livestock and wildlife. The causative agents of all these diseases are known to occur in free-ranging wolves^20^. It would therefore be highly instructive to know which helminth species are circulating within the CEL wolf population. Equally, little is known about protozoan infections in wolves, even though wolves could potentially be the definitive host and vector of microparasite diseases such as neosporosis or sarcosporidiosis, which play a vital role for wildlife, livestock and public health in general^32^.

In this study, we therefore tackled the questions (1) which endoparasite species are circulating within the CEL wolf population, (2) whether these parasites are zoonotic, (3) to which extent wolves may have an epidemiological influence on their local prey species, and (4) if and to what degree the endoparasitic load of an expanding wolf population changes within the first years of resettlement. To address these issues, we apply a variety of molecular tools to identify individual wolves, their helminth and protozoan community retrieved from whole carcasses, as well as cysticerci isolated from their prey. We use this information to characterise the parasite infection status of individual wolves and subsequently test the influence of intrinsic factors such as age, sex and genetic constitution, and extrinsic factors such as population size and geographic origin on parasite diversity in an expanding wolf population. By knowing the genetic identity of most wolf packs of the German part of the CEL population, we could also identify ‘immigrants’ – wolves that were not born in one of the known German packs – and (5) identify the parasite species ‘imported’ by them.

## Results

### Genetic structure of wolf sample

As part of the German national wolf monitoring, we dissected and genotyped 53 carcasses between 2007 and 2014. One common mtDNA control region haplotype, HW01 dominated in the 52 successfully analysed individuals, with the exception of a single HW02 wolf (corresponding to haplotypes w1 and w2 described in other studies^33^). By comparing the 53 microsatellite-based genotypes to the German wolf genotype database (unpublished), 36 wolves could be assigned to packs in Germany and thus were considered ‘native’. The remaining 17 genotypes showed no first-order relationship to known German packs and were thus considered likely to be ‘immigrant’ individuals from Western Poland or the Baltic wolf population.

Subsequent Bayesian population clustering suggested five population clusters (see Supplementary Fig. S1). One individual was assigned to the group of reference samples of the Baltic wolf population (CL87/14 haplotype HW02), and three individuals (CL79/12, CL133/12, CL534/12) showed intermediate genotypes. All other wolves formed a single, distinct CEL wolf cluster, indicating a genetic separation of this newly established population from its Baltic source population. Microsatellite allele frequencies from the CEL wolf population were distinctly different from domestic dog reference samples.

Individual microsatellite heterozygosities ranged between 0.36 and 0.86 with a mean value of 0.6 (SEM = 0.02, 95% confidence limits 0.57–0.63, n = 53).

### Helminth diversity in wolves

Alpha diversity of the helminth population was determined by species richness and the Shannon index – a measure of diversity considering both the number of occurring species and their abundance. Infection with a single helminth species was recorded in 20.8% of the cases. Co-infection occurred most frequently with two species (22.6%), constantly decreasing to three helminth species (20.8%), four species (11.3%), five, six or seven helminth species (3.8% each). Eight helminth species per wolf were only detected once (1.9%), while 11.3% were helminth negative. Mean species richness over all individuals was 2.57 (SEM = 0.26, 95% C.L. 2.03–3.10, n = 53). Helminth species richness in ‘native’ individuals was 2.72 species (SEM = 0.34, 95% C.L. 2.03–3.41, n = 36) and in ‘immigrants’ 2.24 species (SEM = 0.39, 95% C.L. 1.41–3.06, n = 17). Helminth diversity, as measured by the Shannon index, ranged between 0 and 1.35 with a mean value of 0.38 (SEM = 0.06, 95% C.L. 0.27–0.49, n = 51).

**Figure 1:**
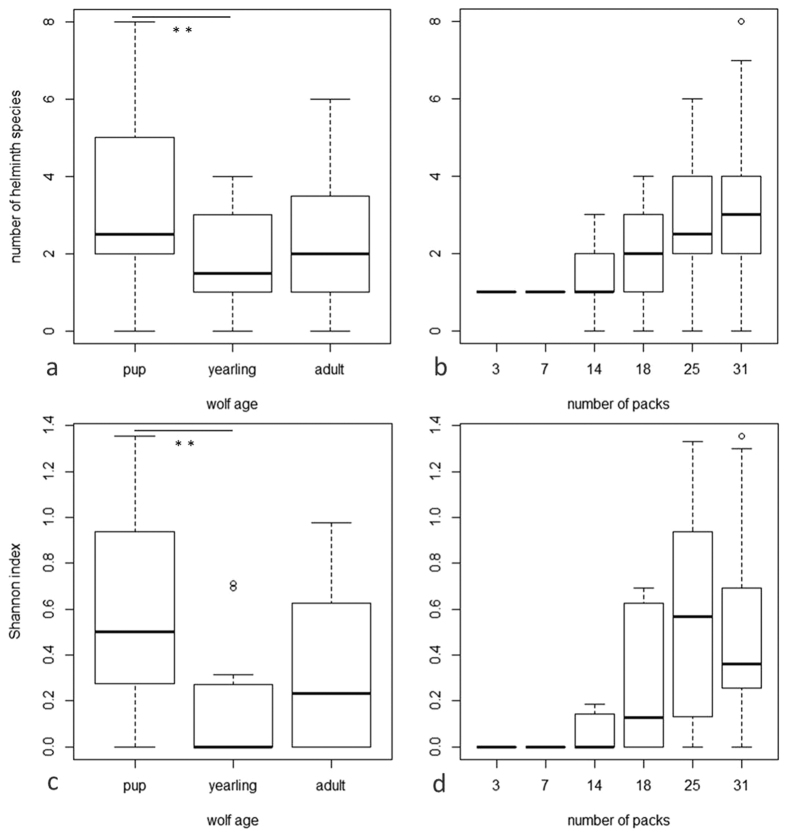
Relevant effectors of helminth species richness and diversity (Shannon index) in wolves from the CEL population. Helminth species richness (**a,b**) and helminth diversity (**c,d**) vary with wolf age significantly decreasing from pups to yearlings (n_pup_ = 21, n_yearling_ = 16, n_adult_ = 14) and increase with wolf population size (n_3packs_ = 1, n_7packs_ = 1, n_14packs_ = 8, n_18packs_ = 11, n_25packs_ = 14, n_31packs_ = 16). Dots represent outliers. Box plot edges depict the quartiles for number of helminths species (**a,b**) and the Shannon index (**c,d**). Whiskers extend to non-outlier extremes. Statistical significance was calculated using a general linear model.

Helminth species richness (general linear model, overall likelihood ratio test, χ^2^ = 23.865, df = 6, p < 0.001, n = 51) significantly increased with population size (F_1,46_ = 14.58, p < 0.001, Fig. 1b) and significantly changed with wolf age category (F_2,46_ = 4.688, p = 0.014, Fig. 1a). Pairwise post-hoc tests indicated that helminth species richness significantly declined from pups to yearlings (p = 0.006). Similarly, helminth diversity (general linear model, overall likelihood ratio test, χ^2^ = 25.967, df = 6, p < 0.001, n = 51) significantly increased with population size (F_1,46_ = 10.77, p = 0.002, Fig. 1d) and significantly changed with wolf age category (F_2,46_ = 5.230, p = 0.009, Fig. 1c). Pairwise post-hoc tests indicated that helminth diversity significantly decreased from pups to yearlings (p = 0.004). Sex, microsatellite heterozygosity, and geographic origin had no significant effect on helminth species richness and diversity.

The genus *Taenia* was the most prevalent and most abundant genus of helminths (0–109 parasites per individual). *Taenia* spp. abundance category (multinomial logistic regression, overall likelihood ratio test, χ^2^ = 22.635, df = 12, p = 0.031, n = 51, Table 1) changed significantly with geographic origin of wolves, with ‘immigrants’ significantly more often showing a high level of *Taenia* abundance than ‘natives’. Sex (p = 0.073) and age (p = 0.079) marginally affected *Taenia* abundance, in that females were more likely to either have high or no *Taenia* abundance than males, and yearlings had lower levels of *Taenia* abundance than pups or adults. Genetic heterozygosity and population size had no influence.

### Helminth fauna and prevalence in wolves

Thirteen helminth species were identified based on 18S rRNA and cytochrome c oxidase subunit I genes (Supplementary Table S1), while in 11% and 8% of the cases the isolated lung and intestinal nematodes could not be determined. Nematodes were the most diverse class (eight species), followed by cestodes (four species), and trematodes (one species). Infestations with the highly zoonotic *Trichinella* species *T. britovi* and *T. spiralis* (in muscular tissue) and with the fox tapeworm *E. multilocularis* were documented in three single cases, each representing a rare species in wolves, with a prevalence of 2%. The cestode *T. krabbei* was the most common (77%) helminth species in wolves, and is therefore considered the core species (by definition > 60% prevalence^34^,^35^) in this population. *T. hydatigena* and *Mesocestoides litteratus* were identified in 15% and 9% of wolves, respectively. *A. alata*, the only trematode, was detected in 53% of all wolves and can therefore be considered a secondary species (by definition 40–60% prevalence^34^,^35^). The three intestinal nematodes *Uncinaria stenocephala* (11%), *Toxocara canis* (11%) and *Toxascaris leonina* (4%) were isolated less frequently than cardio-pulmonary parasites. The two lung nematodes *Crenosoma vulpis* and *Capillaria aerophila* were found in 25% and 15% of all cases. *C. plica* was isolated from the urinary bladder of 25% of all wolves.

**Figure 2:**
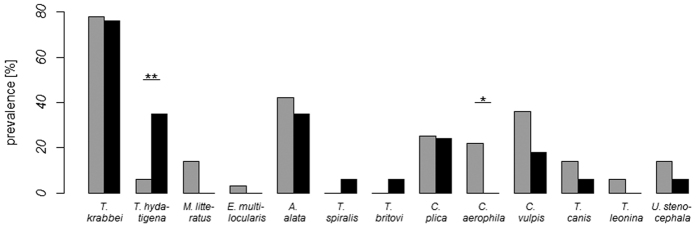
Helminth prevalence of CEL wolves in relation to their geographic origin. ‘Native’ wolves (grey bars) had a significantly lower prevalence of the tapeworm *T. hydatigena* (p = 0.010) and a significantly higher prevalence of the lung nematode *C. aerophila* than ‘immigrants’ (black bars) (p = 0.044). Statistical significance was calculated using the Fisher’s exact test.

In total, 89% of investigated wolves carried endoparasites. Differences in the helminth fauna of all 53 individuals as a function of their geographic origin are depicted in Fig. 2. The cestode *T. hydatigena* occurred significantly less frequently in ‘natives’ born in Germany (3%) than in ‘immigrants’ (37%) (Fisher’s exact test, p = 0.010, 95% C.L. 1.33–101.54, n = 53). In contrast, ‘native’ wolves had a significantly higher prevalence of the lung nematode *C. aerophila* (24%) than ‘immigrants’ which were not infected with this helminth at all (Fisher’s exact test, p = 0.044, 95% C.L. 0.00–1.12, n = 53). There were no significant prevalence differences for other helminths between immigrated and native individuals.

### Sarcocystis fauna and diversity

**Figure 3:**
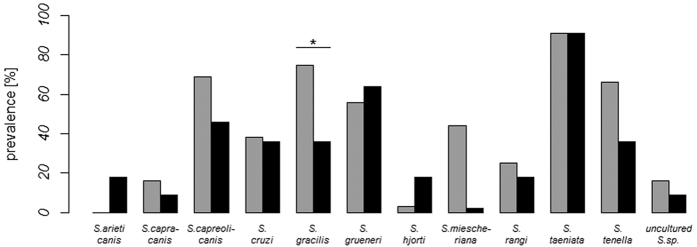
*Sarcocystis* spp. prevalence of CEL wolves in relation to their geographic origin. ‘Native’ wolves (grey bars) had a significantly higher *S. gracilis* prevalence than ‘immigrant’ wolves (p value = 0.031) (black bars). Statistical significance was calculated using the Fisher’s exact test.

Each of the 15 used primer sets successfully amplified *Sarcocystis* spp. DNA on the integrated fluidic circuit. For brevity we use the term ‘species’ to refer to ‘operational taxonomic units’ (OTUs)^36^ from our molecular identification approach. Metabarcoding of the *Sarcocystis* spp. 18S rRNA gene revealed the presence of at least 12 different potential species of the genus *Sarcocystis* with a total prevalence of 95% (n = 43). The most prevalent OTU had an 18S rRNA sequence identical to *S. taeniata* (91%), *S. gracilis* (65%), *S. capreolicanis* (63%), *S. grueneri* (58%) and *S. tenella* (58%). Less than half of the wolf population was infected with *S. miescheriana* (40%), *S. cruzi* (37%), *S. rangi* (23%), *S. capracanis* (14%), *S. hjorti* (7%), and *S. arieticanis* (5%). In 14% of all cases, the isolated sequence was assigned to an undetermined *Sarcocystis* species. *S. gracilis* was the only species that occurred with a significantly higher prevalence in ‘native’ wolves than in ‘immigrants’ (Fisher’s exact test, p = 0.031, 95% C.L. = 0.03–1.02, Fig. 3). Sarcocystis species richness could not be predicted from sex, age, microsatellite heterozygosity, geographic origin, and population size (general linear model, overall likelihood ratio test, χ^2^ = 5.525, df = 6, p = 0.478, n = 43).

### Cysticercoses in ungulate intermediate hosts

In both study areas, *T. krabbei* and *T. hydatigena* were the only metacestodes detected in three of the four ungulate species (Table 2). In our limited sample of fallow deer, no individual was infected with any kind of cysticercus, while in red deer and roe deer *T. krabbei* and *T. hydatigena* prevalences were low, ranging from 0% to 6.1%. Wild boar were solely infected with Cysticercus tenuicollis. There was no significant cyst prevalence difference between wolf territories and the control area for a single ungulate species (Table 2). Using a general linear model (overall likelihood ratio test, *Χ*^2^ = 10.219, df = 6, p = 0.069, n = 440) we were not able to show a significant effect of wolf presence on the cysticercosis prevalence across all ungulates. However, there was still a trend (p = 0.084) indicating that ungulates from wolf areas have a marginally higher cysticercosis prevalence.

## Discussion

The recent recolonisation of large carnivore populations in Europe is a remarkable success of conservation efforts based on legislative decisions, increased public awareness, and scientific knowledge^18^. Wolves had been eradicated from Central Europe for about a century. The CEL wolf population has grown from one pack in the year 2000 to approximately 60 packs by 2015, and continues to expand and increase^17^,^37^. We used this unique quasi-experimental environment to investigate how endoparasite diversity is affected by founder events, how prey populations interact in parasite transmission to predator hosts and vice versa, and which intrinsic and extrinsic factors control parasite diversity. To address these questions, we dissected entire wolf carcasses, applied classical and molecular genetic techniques to identify individuals and their helminth community, and used a metabarcoding approach to analyse whole gut sediments to screen for protozoan parasites. With this study we intended to generate appropriate evidence to clarify potential public health issues, which frequently arise during recolonisation events of large carnivore populations.

We applied mtDNA sequencing to haplotype wolf individuals and to identify their geographic origin. This revealed two haplotypes, HW01 and HW02, which are widespread across Europe^33^ and commonly found in the CEL wolf population, including German wolves (ref. 16; German wolf genotype database, unpublished). Microsatellite-based structure analysis suggested that all but one carcass were likely to come from the CEL wolf population, which is clearly differentiated from the Baltic, Carpathian, and Alpine populations. Three individuals could not be clearly assigned to either the CEL or the Baltic wolf population, suggesting the existence of a contact zone or the possibility of long-distance dispersal with successful admixture. Individual CL87/14 was identified as the second male introducing haplotype HW02 to Germany and thus this individual provides the only obvious case of gene flow from an adjacent source population in our dataset. Using the German genetic wolf database, almost three-quarter of the wolf carcasses were assigned to German packs, providing evidence of their native origin. Seventeen wolves could not be assigned to any genetically known German pack and were thus considered most likely to be immigrants from Western Poland.

To investigate the endoparasite fauna in these wolves we avoided – commonly conducted but inadequate – parasitological scat analyses, which often underestimate parasite diversity^38^, owing to intermittent egg shedding, biases towards hermaphroditic or female parasites, and limitations towards species that excrete their eggs through the intestine. But still our approach of isolating parasites from dead and partially decomposed wolves implies a minor drawback. While we were able to collect data on helminth species richness using molecular techniques in each case, counting of cestodes was not possible in 22 cases due to decomposition. To overcome this problem when calculating the Shannon index, we used mean values depending on the infestation level and based the statistical model regarding helminth diversity on these approximate values.

Furthermore, a sample size of 53 wolves might appear to be relatively small, but despite the limited availability of carcasses it is still a well-represented sample considering the current population size of approximately 39 known wolf packs in Germany. Based on these 53 individuals, we investigated the drivers of parasite species richness and diversity in wolves during their recolonisation of Central Europe by using individual characteristics (age, sex, genetic heterozygosity, parasite load) as well as geographic background data (‘native’ versus ‘immigrant’) of each individual. Interspecies studies in mammals have shown that parasite richness and diversity in free-ranging wildlife can generally be influenced by biogeographical, ecological, immunological, life-history traits, and individual characteristics^3^,^4^,^5^,^6^. In concordance with previous intraspecific parasite ecology studies in European wolves^10^,^39^,^40^, we analysed the correlation of sex, age, geographic origin, and genetic constitution with parasite diversity in wolves, and additionally investigated the effect of a growing host population size – given the circumstances of the current CEL wolf population expansion. None of the previous studies found an effect of sex^10^,^40^ or geographic origin^10^,^40^, while the prevalence of particular helminth species was correlated with age^10^,^39^ and year/season of death^39^. In contrast to these studies, we analysed the correlation of host parameters with parasite alpha diversity instead of single helminth species, and confirm that age significantly affected the level of parasite alpha diversity, which is also consistent with helminthological findings in domestic dogs^41^. Helminth diversity, parasite species richness and *Taenia* spp. abundance decreased from pups to yearlings, then tended to increase from yearlings to adults, suggesting two separate processes to be responsible for these changes. Age-intensity relationships in helminth disease etiopathology have also been described in other species^42^,^43^,^44^,^45^ but the interpretation of such data currently remains vague, though opening room for speculation about adaptive immune processes during early life and posterior accumulation effects.

As wolves – and potentially their parasites – had been eradicated from Central Europe for more than a century, we investigated the effect of an increase in wolf population size on parasite alpha diversity. Helminth diversity and helminth species richness increased with the annually growing number of wolf packs, but not Sarcocystis species richness or Taenia abundance. While density-dependent effects of parasite diversity have been repeatedly discussed in cross-species approaches^4^,^5^,^46^, host population size has – to our knowledge – not been previously considered in an intraspecific study focussing on wolves in Europe. Our work provides principal evidence that wolf helminth diversity increases during wolf population expansion, indicating that density-dependent parasite transmission amongst conspecifics and between wolves and their prey might play a major role in this carnivore. As wolves share their parasites with other predator and prey species, it is currently not clear to what extent alternative carnivore hosts transmit typical canine endoparasites in the area currently occupied by the German wolf population. For Central Europe in particular, anthropogenic factors such as tourism and hunting are likely to influence endoparasite communities of wildlife, since domestic dogs, particularly hunting dogs, share a similar diet with wolves and may serve as an additional parasite reservoir.

‘Immigrants’ had a higher abundance of *Taenia* cestodes than ‘residents’, potentially indicating an effect of the geographic origin in terms of either former habitat, *Taenia* metacestode infection in prey in the local habitat, or potential immunogenetic differences between ‘immigrants’ and ‘natives’. Individual heterozygosity as measured by microsatellites did not correlate with parasite alpha diversity in our wolves, although heterozygosity has been associated with individual and population fitness and stress resistance, including parasite and disease susceptibility^47^. The mean heterozygosity of 0.6 in our wolves was lower than that of other European populations, but higher than in some small populations with a recent bottleneck history such as the Italian one^48^. This moderate level in combination with the fact that microsatellite markers may not appropriately reflect functional or genome-wide heterozygosity^49^ might explain the lack of a significant association with parasite diversity and richness. The relatively low number of non-coding genetic markers might not be linked to functional immunogenetic loci, so potential associations between genome-wide heterozygosity and parasite load would become indistinct. Such loci play a fundamental role for pathogen resistance and are important indicators in evolutionary ecology and conservation^50^. Hence, genetic diversity of loci under balancing or positive selection, such as the major histocompatibility complex^51^ should be studied to evaluate the CEL wolf population’s genetic potential to cope with parasites and reveal whether a founder effect has created a potentially impaired immune competence.

When analysing helminth communities and prevalence in wolves relative to their geographic origin, we found further implications potentially arising from immunogenetic or habitat effects. The lung nematode *C. aerophila* was exclusively found in ‘native’ wolves, while ‘immigrants’ had a significantly higher prevalence of *T. hydatigena*, so ‘immigrants’ can be considered importing this cestode into German wolf territories. Despite some ungulate intermediate hosts of *T. hydatigena* being uncommon in Germany, e.g. moose (*Alces alces*)^52^, our cysticercosis screening in German ungulates demonstrates the presence of Cytsticercus tenuicollis in different parts of Germany (see below).

General helminth prevalence in our sample of wolf carcasses was 89%, which is similar to what several other studies found in wolves from the Baltic population (see Supplementary Table S3 for literature comparison of all species). With 13 genetically distinguishable helminth species and a mean of 2.57 ± 0.26 (SEM) species per individual, our sample had a significantly lower helminth species richness than Latvian^10^ or Polish^38^ wolves. At least 11 out of 13 isolated species have also been diagnosed in their Eastern relatives from the Baltic population. Presumably, the founders of the CEL wolf population had introduced a subset of the ‘original’ helminth community of the Baltic population, while at the same time intermediate hosts of some parasites are potentially absent in Central Europe such as moose or European bison (*Bos bonasus*). Our study suggests that parasite prevalence and diversity in the CEL wolf population will increase over time with ongoing expansion and immigration of new individuals.

Among the 13 helminth species, the three highly zoonotic parasites *T. spiralis, T. britovi* and *E. multilocularis* occurred in one case each (prevalence 2%). Hence, wolves play a minor role as reservoir of *Trichinella* larvae. Likewise, their role as vector and reservoir of *E. multilocularis* in Europe is insignificant compared to foxes, which occur in higher numbers and can reach a local prevalences between 0% and 60%^53^.

Further results of our literature comparison of helminth prevalences with Baltic wolves sampled in Poland, Latvia, Estonia and Belarus are depicted in Supplementary Table S3. Most likely, significant differences can be explained by (1) higher or lower general prevalences of the particular parasite in the alternative habitat, (2) higher or lower prevalence of intermediate or additional definitive hosts in that habitat or (3) differing wolf diet and thus avoidance of the particular parasite.

An illustrative example where all hypotheses could be tested is the detection of the trematode *A. alata* which occurred more frequently in German wolves than in Belorussian ones, but less often than in Latvian and Estonian wolves, where it is the most frequent helminth. Since *A. alata* infection in carnivores depends on the consumption of infected wild boar meat, prevalence differences might either occur due to differing regional trematode abundances resulting from (1) varying environmental conditions for parasite development, (2) varying abundance of primary (snails) and secondary hosts (wild boar), or (3) due to regional differences in wolf diet.

*Taenia* represents another important helminth genus in our wolf sample that requires a two-host-cycle (herbivore/omnivore and carnivore). Wolves from the Baltic population were infected with a higher diversity of *Taenia* species than our wolves, in which *T. krabbei* (prevalence 77%) and *T. hydatigena* (prevalence 15%) were the only two detected species. This loss of *Taenia* spp. richness suggests that the founders of the CEL wolf population started with a reduced parasite community and/or that German wolves fed on a lower diversity of prey and therefore acquired fewer cestode species. This in turn might change during a longer presence of wolves as definitive hosts altering transmission dynamics.

Our cysticercosis screening in wild ungulates was intended to assess whether metacestode prevalence differed in the four main prey species of wolves in Germany between areas with and without wolves. Both detected cestode species *T. krabbei* and *T. hydatigena* are known to cause cysticercoses in wild and domestic ungulates. Contrasting our hypothesis, metacestode infection rates did not differ significantly between the two study areas, but still we found a trend of prevalences being slightly higher when wolves are present. Given the relatively low metacestode prevalence in both study areas, it was not feasible to sample an appropriate number of individuals in order to increase the statistical power of the analysis. Furthermore, it is not only wolves that shed their parasites into the environment. So the role of alternative definitive hosts such as domestic dogs, red foxes or racoon dogs must not be underestimated and should be investigated in future studies before final conclusions can be drawn.

Unfortunately, comprehensive cysticercoses data from Central and Eastern European wild ungulates are scarce. However, a recent Danish study reported the reoccurrence of *T. krabbei* cysticerci in roe deer after more than 60 years of absence in this species^25^ and suggested that wolves may be responsible, since a *T. krabbei* infected individual had been documented in the same area^54^. Underlining the need to evaluate the role of alternative hosts, notably higher *T. krabbei* metacestode prevalences were reported during the 1970s, with 33% in roe deer and 19% in red deer^55^, even though wolves have not been resident in Hungary (see Supplementary Table S4 for literature comparison). *T. hydatigena* prevalence was also significantly higher in all four ungulate species compared to Germany. Hydatid disease caused by *E. granulosus* was not detected in German ungulates but found at remarkably high rates in wild boar and red deer in Eastern Europe^55^,^56^.

We could not include skeletal muscle tissue of ungulates in our screening, so the only muscular tissues analysed macroscopically were tongue, heart and diaphragm. This might have made us underestimate *T. krabbei* prevalence and miss the zoonotic *A. alata* mesocercariae and *Trichinella* larvae in the diaphragm of wild boars. Nevertheless, the low species-specific total *Taenia* prevalences between 0% and 5% (see Supplementary Table S4) suggest that larval cestode infections have a minor health impact on the analysed ungulate populations in Germany.

The protozoan parasite *Sarcocystis* is known to cause sarcocystosis and sarcosporidiosis in its intermediate and definitive hosts, respectively. Identification of sarcocysts from the intermediate host’s musculature has been conducted for decades, while identifying *Sarcocystis* sporocysts from the definitive host usually requires complex infection experiments^57^ or laboratory methods^58^, and has therefore been rarely performed, especially in wild large carnivores. While morphological studies in Europe have not provided any data on *Sarcocystis* prevalence in wolves, *Sarcocystis* spp. prevalence in Canada ranged from 38%^59^ to 100%^60^. Using metabarcoding on whole gut sediments to analyse *Sarcocystis* spp. diversity in free-ranging wolves, we found that 95% of our wolves were *Sarcocystis* positive.

Technically, our metabarcoding approach enables us to determine ‘operational taxonomic units’ as clusters of similar sequences^36^. For brevity, we use the term ‘species’ instead, accepting the limitations of our method. The species identified via database entries – as sharing highest sequence similarity with our data – have been previously described from various wild and domesticated ungulate intermediate hosts. *S. taeniata* and *S. hjorti* are known in moose^61^,^62^ and red deer^63^,^64^, while *S. capreolicanis* and *S. gracilis* usually occur in roe deer^62^. In our sample of wolves *S. gracilis* was significantly more prevalent in ‘natives’ than ‘immigrants’. As for helminths, such differences could occur due to potential habitat, immunogenetic or diet differences. These findings are independent of our potentially limited species resolution capacities, and whether or not a particular parasite strain with prevalence differences is granted species status.

*S. grueneri* sarcocysts develop in reindeer^65^, red deer^66^ and fallow deer^67^; the latter two being the most likely source of infection for our wolves. *S. miescheriana –* known from wild boars^68^ and domestic pigs^69^ – had a prevalence of 40% in German wolves, consistent with the fact that wild boars contribute 18% of biomass to the German wolf diet^23^. In contrast, sequences sharing highest similarity with *S. rangi, S. tenella, S. arieticanis, S. cruzi*, and *S. capracanis* were detected more often than expected, since wolves usually do not commonly prey on reindeer^65^, mufflon^70^, domestic sheep^62^, cattle^71^ or goats^72^, respectively. This discrepancy suggests either a lack of resolution in the sequenced gene fragment and that those sequences represent different – yet to be described – species, or that these described species have a broader intermediate host spectrum than previously thought.

While the incidence of emerging infectious diseases has increased in recent decades^73^, the presence and impact of wildlife has often been neglected. Wild carnivores may play a major role for the distribution of infectious disease and different host species sharing the same parasites may have an epidemiological influence on each other which is often of complex nature and hard to capture when only focusing on one target species. Our findings suggest that wolves from Central Europe currently have a minor relevance as reservoir of zoonotic parasites. Since we also show that parasite alpha diversity changes with growing wolf population size, the situation might best be described as being in a dynamic state. Thus, it might be useful to implement an endoparasite screening as a future monitoring tool to ease public and veterinary health concerns, since parasite life cycles are complex and some are flexible and may therefore change with time and expanding host population range. In fact, especially domestic dog owners and hunters in wolf habitats are likely to benefit from our findings, helping to make well-informed decisions on anthelminthic dog treatment and ungulate meat hygiene. Since hunters periodically feed their dogs with potentially infected meat, our results suggest that a routine anthelminthic treatment of hunting dogs would be highly advisable as recommended by the European Scientific Counsel for Companion Animal Parasites (ESCCAP).

## Material and Methods

### Sample collection

Between 2007 and 2014, we examined 53 wolf carcasses, collected as roadkill or poached, originating from five federal states in North and East Germany (50°10′–54°54′N and 6°41′–15°2′E) for endoparasites. Depending on recovery conditions (mostly time period between death and recovery of the carcass, outside temperature) we received the carcasses in different states of decomposition. Wolf sex and age category were determined by computed tomography and during necropsy by two specialised veterinarians for radiology and pathology. Age was estimated by assessing body size and mass, tooth replacement, tooth abrasion, state of thymus involution, state of reproductive organs as well as size and state of growth plates. Age class estimates were furthermore cross-checked and validated with the German wolf monitoring database (www.wildtiergenetik.de) by knowing the individual genetic identity. According to the joint monitoring standards for the CEL wolf population, day of birth was set to the 1st of May by default^17^. Individuals were considered as ‘pup’ within their first year of life, ‘yearlings’ within their second year, and ‘adults’ were older than 2 years. Helminths were isolated from all inner organs by conventional parasitological dissection^74^. When carcasses were fresh, we were able to recover and count all helminths (n_wolves_ = 29). Taenia spp. abundance was additionally classed into the categories ‘no’, ‘low’ or ‘high’ load. However, when carcasses were in an advanced stage of decomposition, cestodes were partially degraded and could therefore not be counted, but still their abundance was estimated using the above mentioned categories (n_wolves_ = 22). Detection of *Trichinella* larvae from muscular tissue was carried out by the National Reference Laboratory for *Trichinella* (Federal Institute for Risk Assessment, Berlin, Germany). We collected intestinal protozoa by washing and sieving the whole gut to eliminate food remains and collected the flow-through (n_wolves_ = 43).

Between 2012 and 2014, we collected 440 individuals of ungulates shot during hunts and screened their inner organs for cysticerci. To do so, we inspected the surface (including connective tissue) of lung, heart, diaphragm, spleen, liver, kidneys, intestines and mesentery, and sliced the tissues into 1 cm thin layers to inspect their interior parts. Isolated metacestodes were stored at −20 °C until DNA extraction. We compared cysticercosis prevalence in these ungulates between wolf territories (German federal states of Brandenburg and Saxony, 50°10′–53°33′N and 11°14′–15°2′E) and a control area where no territorial wolves were known at the time of sampling (German federal state of Schleswig-Holstein, 53°20′–54°55′N and 8°36′–11°7′E). The screening comprised fallow deer (*Dama dama*, n_wolf territories_ = 7, n_control_ = 28), roe deer (*C. capreolus*, n_wolf territories_ = 105, n_control_ = 72), red deer (*C. elaphus*, n_wolf territories_ = 82, n_control_ = 20), and wild boar (*S. scrofa*, n_wolf territories_ = 88, n_control_ = 38).

### DNA extraction

For wolf genotyping, ethanol-preserved tissue samples were extracted using the QIAamp DNA Blood & Tissue Kit (Qiagen, Hilden, Germany) following the instructions of the manufacturer. Helminth DNA was extracted from minced and proteinase K digested tissues using phenol-chloroform-isoamyl alcohol and a standard protocol (Carl Roth, Karlsruhe, Germany). Extraction success and DNA concentrations were determined using the NanoDrop^®^ 1000 spectrophotometer (Thermo Fisher Scientific Inc., Waltham, Massachusetts, USA).

Wolf protozoan (microparasite) DNA was extracted from 500 μl of pelleted intestinal filtrate suspended in 700 μL Buffer SL2 using the NucleoSpin^®^ Soil Kit (Macherey-Nagel, Düren, Germany) according to the manufacturer’s protocol. Extraction success and DNA concentrations were determined using the Qubit^®^ 2.0 Fluorometer (Invitrogen, Carlsbad, California, USA).

### Microsatellite PCR and sequencing

We used 13 variable microsatellites and two sex markers (DBX6 and DBY7^75^) to assess relatedness and origin of the wolves. Markers CPH5^76^, FH2001, FH2010, FH2017, FH2054, FH2087L, FH2088, FH2096, FH2137, FH2140 and FH2161^77^, vWF^78^, and PEZ17^79^ were amplified in three 10 μl multiplex PCRs containing HotStarTaq Master Mix (Qiagen, Hilden, Germany), 0.2 μM of each primer, 2 ng BSA and ~5 ng genomic DNA. PCR started with initial denaturation at 95 °C (15 min), 4 cycles of 94 °C (30 s), 60 °C (90 s) and 72 °C (60 s); another 5 cycles of 94 °C (30 s), 58 °C (90 s) and 72 °C (60 s), 5 cycles of 94 °C (30 s), 58 °C (90 s) and 72 °C (60 s); another 5 cycles of 94 °C (30 s), 54 °C (90 s) and 72 °C (60 s), 25 cycles of 94 °C (30 s), 50 °C (90 s) and 72 °C (60 s), and a final elongation at 72 °C (30 min). PCR products were size-measured on an ABI 3730 DNA Analyzer (Life Technologies, Carlsbad, California, USA) and scored using GeneMarker v1.90 (SoftGenetics, State College, Pennsylvania, USA) by comparison to LIZ600 as an internal size-standard. For mitochondrial DNA control region sequencing, primers L15995^80^ and H16498^81^ were used. PCRs were performed in 15 μl containing 3 mM MgCl_2_, 1.5 μl 1 × PCR buffer, 0.13 mg/μl BSA, 0.2 mM dNTPs, 0.333 μM of each primer, 1 U *Taq* polymerase (New England Biolabs Inc., Ipswich, Massachusetts, USA), and 3 μl DNA extract. PCR protocol started with initial denaturation at 95 °C (3 min), 35 cycles of 94 °C (30 s), 54 °C (30 s) and 72 °C (60 s), and a final elongation of 72 °C (10 min). PCR products were purified using ExoSAP-IT (USB Corporation, Cleveland, Ohio, USA) following the manufacturer’s protocol. Sequencing was carried out on an ABI 3730 DNA Analyzer (Life Technologies, Carlsbad, California, USA). Sequencing results were analysed in Geneious v7.1.9 (Biomatters Ltd, Auckland, New Zealand) and compared to sequences deposited in the NCBI database.

### Macroparasite PCR and sequencing

Cestodes, trematodes and intestinal nematodes were identified targeting a 450 bp fragment of the cytochrome c oxidase subunit 1 gene (cox1). The cox1 PCR was carried out using the primer set JB3 (5′-TTTTTTGGGCATCCTGAGGTTTAT-3′) and JB4.5 (5′-TAAAGAAA GAACATAATGAAAATG-3′) previously described^82^,^83^. Additionally, we used the primers 18S_965F (5′-GGCGATCAGATACCGCCCTAGTT-3′) and 18S_1573R (5′-TACAAAGGGCAGGGACGTAAT-3′)^84^,^85^ to amplify and sequence a 620 bp fragment of the 18S rRNA gene to identify cardiopulmonary and urinary helminths.

PCRs were performed in an epGradient S thermocycler (Eppendorf, Hamburg, Germany) and had a total volume of 25 μl per sample, including 1 μl DNA template. The reactions contained 1 × FastStart High Fidelity Reaction Buffer without MgCl_2_, 0.2 mM dNTPs, 2.5 mM MgCl_2_, 0.2 μM of each primer (cox1) or 1 μM of each primer (18S rRNA) 0.4 μg/μl BSA (only cox1 PCR) and 0.5 U FastStart High Fidelity Enzyme Blend (all components from Roche, Basel, Switzerland). PCRs were run in 40 cycles, starting with an initial denaturation step at 95 °C (10 min), and ending with a final elongation step at 72 °C (10 min). Thermal cycling of the cox1 PCR took place as follows: 95 °C (45 s), 55 °C (45 s), 72 °C (60 s). Thermal cycling of the 18S rRNA PCR took place as follows: 95 °C (30 s), 53 °C (30 s), 72 °C (60 s).

Of each helminth PCR product, we purified 1 μl using 1 U FastAP Thermosensitive Alkaline Phosphatase (Thermo Fisher Scientific, Waltham, Massachusetts, USA) and 3 U Exonuclease I (Thermo Fisher Scientific, Waltham, Massachusetts, USA) due to the manufacturer’s protocol. Sequencing PCR and clean-up were performed under standard conditions using the BigDye^®^ Terminator v3.1 Cycle Sequencing Kit and the BigDye Xterminator^®^ Purification Kit (Life Technologies, Carlsbad, California, USA) before loading them on the Applied Biosystems^®^ 3130xl Genetic Analyzer (Applied Biosystems, Foster City, California, USA).

### Microparasite library preparation and sequencing

The eukaryote 18S rRNA gene is typically grouped into nine variable regions V1–V9 suitable for diversity studies of several taxa^86^. In order to design primers that flank the most variable regions of the *Sarcocystis* spp. 18S rRNA gene (see Supplementary Fig. S2), we used primer3 version 0.4.0^87^ and oligonucleotides previously described. Sensitivity of oligonucleotide binding to the targeted Sarcocystis spp. sequences was assessed using the tool TestPrime 1.0 in the Silva web interface^88^. Metabarcoding PCR on an integrated fluidic circuit (48.48 Access Array™ IFC by Fluidigm, San Francisco, California, USA) was performed as a duplicate experiment using 15 ng DNA from wolf intestinal contents and the 15 primer sets (Supplementary Table S2). As the assay is limited to 48 wells, we decided to use 43 wolf samples and included 5 quality controls into each run. All amplification and barcoding PCR steps, as well as library preparation steps were carried out according to the manufacturer’s user guide (Access Array™ System for Illumina Sequencing Systems, Chapter 6, Fluidigm, San Francisco, California, USA). After running the 48.48 Access Array IFC, we used a 10-fold dilution of the harvested PCR products to perform the barcoding step using the Access Array Barcode Library for Illumina Sequencers - 384 (Single Direction) (Fluidigm, San Francisco, California, USA). Post-PCR quality control steps included amplicon quality and length check using the 2200 TapeStation Instrument with D1000 ScreenTapes and D1000 Reagents (Agilent Technologies, Santa Clara, California, USA). Afterwards, PCR fragments between 400 and 1000 bp were purified by PippinPrep using the 1.5% agarose DNA gel cassettes (Sage Science Inc., Beverly, Massachusetts, USA). Then, we pooled the samples and authorized a next-generation sequencing service using the MiSeq Reagent Kit v3 on the MiSeq sequencing system (Illumina, San Diego, California, USA).

### Bioinformatics

In order to identify the isolated macroparasites from wolves and ungulates at the species level, we merged the Sanger sequenced forward and reverse reads using the programme SeqMan implemented in Lasergene (DNASTAR Inc., Madison, Wisconsin, USA). Additionally, sequences and the corresponding electropherograms were verified by eye, and sequences corrected manually where necessary. Subsequently, we searched for these sequences in the GenBank nucleotide collection from the National Center for Biotechnology Information (NCBI) using BLAST^®^^89^ and stored the best hit species and the alternative species as a table (Supplementary Table S1). If results were not distinctly clear, we used further information of alternative genetic markers, helminth morphology, and organ of isolation to determine the most likely species.

In order to identify gastrointestinal microparasites from the Illumina metabarcoding data set, we first stratified sequencing reads by amplicon searching for fully identical matches to target specific primer pairs, starting exactly at the first sequence position (behind the removed adapter sequences) in both forward and reverse reads. This resulted in two types of sequence data: (1) Amplicons shorter than 500 bp with overlapping forward and reverse reads. Those were merged using FLASH version 1.2.8^90^. (2) Amplicons larger than 500 bp without forward and reverse read overlap (because of Illumina MiSeq Reagent Kit v3 maximum read length of 300 bp) were not merged, but quality trimmed with Trimmomatic version 0.36^91^. We searched remaining reads in an apicomplexan subset of the NCBI nucleotide database using BLAST^®^^89^. Only hits with a biunique best bit score to one species were further processed and we applied a criterion of 98% identity for the whole length of the query to assign species names. Additionally, a minimum hit length criterion of 200 bp for trimmed reads and 300 bp for merged reads was applied. Thus, our OTUs can be considered clusters of at least 98% sequence identity with respective database sequences over the whole amplicon.

### Statistics – wolf genetics

Bayesian population clustering implemented in Structure software version 2.3.4^92^ was used to test for population origin and potential domestic dog introgression of the 53 wolves. Genotypes of our wolf samples were run together with a set of randomly picked 22 wolf genotypes collected during the German state-based genetic wolf monitoring, reference genotypes from 39 domestic dogs, 16 wolves from the Baltic^18^, 15 wolves from the Carpathian^18^, and 16 wolves from the Alpine region^18^, available from our internal genetic reference database for German wolf monitoring (www.wildtiergenetik.de). Ten independent runs were performed with a K from 2 to 8, a burn-in of 200,000 and 500,000 Markov chain Monte Carlo iterations. We applied an admixture model with correlated allele frequencies. The most probable number of populations was determined based on the second order rate of change of the likelihood^93^ using the web-based programme Structure Harvester version 0.6.94^94^. To compute the optimal assignment to the individual clusters for every individual, the cluster output from the independent runs was permuted by Clumpp version 1.1.2^95^ using the ‘Greedy’ algorithm for aligning replicates.

To reconstruct genetic relatedness we compared all individual genotypes to our internal wolf reference database with >350 individual wolf genotypes, covering most German packs. Genotypes that could be assigned to packs in Germany were considered ‘native’, whereas those with no first-order relationship to a known German pack were considered to be ‘immigrants’. Reconstruction of genetic relatedness was done manually by direct genotype comparison, occasionally supported by use of Coancestry software version 1.0.1.5^96^. Individual heterozygosity was calculated in GenAlEx version 6.5^97^.

### Statistics - parasite diversity

In order to investigate host-parasite interactions, we calculated species richness as the number of endoparasite species, and species diversity using the Shannon index to account for the number of species and their abundance in each individual wolf. We chose to analyse species richness because our methodological approach allows us to extract this information from both presented datasets on helminths and *Sarcocystis*. Moreover, being the most commonly used measure of biodiversity^98^, species richness is easy to compare with available wolf parasite literature^10^,^38^,^99^,^100^ (Supplementary Table S3). Still, the deductions it allows are rather limited to environmental/geographical information in a sense of “parasite availability in a certain habitat”. In this study, we indirectly address such questions by analysing the effect of ‘wolf population size’ and ‘wolf geographic origin’. However, we also included the Shannon index into our analyses, as it accounts for heterogeneities within the parasite community that might potentially be driven by individual host characteristics such as immune capacities which we indirectly intend to correlate by investigating the effect of e. g. ‘wolf genetic heterozygosity’ or ‘wolf age’.

The Shannon index was calculated only for helminths but not protozoan parasites because quantitative measures of *Sarcocystis* presence were not available. Even for helminths, an accurate count of cestodes was not possible in the case of 22 wolf carcasses as they were recovered in an advanced stage of decomposition and the retrieved tapeworms were often highly rotten and fragile. We therefore proceeded as follows: For 51 wolves, *Taenia* spp. abundance could be classified into the three categories ‘no’, ‘low’ and ‘high’ abundance during dissection. We then calculated the means for each category from those wolves where a count was possible and used these as a quantitative estimate of *Taenia* spp. abundance for the 22 wolves with missing *Taenia* spp. count data to calculate a Shannon diversity index for them.

We tested the influence of wolf sex, age, microsatellite heterozygosity, geographic origin of the individual, and wolf population size as defined in Table 3 on helminth and *Sarcocystis* species richness and helminth diversity using general linear models, and *Taenia* spp. abundance using a multinomial logistic regression. In preliminary exploratory analyses we had checked for but found no effect of body mass and carcass recovery location and therefore excluded both predictors from the final analysis. We also excluded year of carcass finding as a high general variance inflation factor (GVIF = 31.97, df = 5) indicated strong collinearity with the predictor population size. For the multinomial logistic regression we report the global summary of the effect of each predictor on the probability of occurrence of each *Taenia* abundance category. We tested the effect of wolf presence on ungulate metacestode infection status using a general linear model and added ungulate species and cestode species as predictors to control for potential species-specific differences (definition of variables see Table 3).

For each multinomial logistic regression, we used log-likelihood ratio tests and information criteria, the Akaike Information Criterion (AIC) and the quasi-likelihood Information Criterion (AICqh) introduced by Hannan and Quinn^101^, to check whether the full model was superior to an intercept-only or a reduced model. Models were considered similar, if differences in AIC were less than 2.5, and preferable, if the difference exceeded 6.0^102^. We also report AICqh values, since they can be of interest in the case of substantial dispersion of data. The significance of each predictor variable was assessed as the marginal contribution of each parameter to the full model by subtracting from the full model the log-likelihood of a second model with each specific predictor removed and testing the difference against a chi-square distribution with the appropriate degrees of freedoms (see refs 103 and 104).

The significance threshold of tests was fixed at 5% and all tests were two-tailed. Statistical analyses were performed in Systat 13 (Systat Software Inc., Richmond, VA, USA) and R version 3.2.1^105^. The Shannon index was calculated using R package *vegan* version 2.3-0^106^. Possible co-linearity of predictor variables was tested with R package *car* version 2.0-26^107^. Multinomial logistic regression was performed in Systat 13. Overall likelihood ratio of the general linear models and multinomial logistic regression was tested using R package *lmtest* version 0.9–34^108^. Pairwise post-hoc comparison was performed with R package *mulcomp* version 1.4–5^109^.

## Additional Information

**How to cite this article:** Lesniak, I. *et al*. Population expansion and individual age affect endoparasite richness and diversity in a recolonising large carnivore population. *Sci. Rep.* **7**, 41730; doi: 10.1038/srep41730 (2017).

**Publisher's note:** Springer Nature remains neutral with regard to jurisdictional claims in published maps and institutional affiliations.

## Supplementary Material

Supplementary Material

Supplementary Table S1

## Figures and Tables

**Table 1:** Multinomial logistic regression of predictors affecting the chance of being in a given *Taenia* abundance category.

Predictor	Direction of effect on chance of *Taenia* abundance being in a given category*			Df	G	p	AIC	ΔAIC	AIC_qh_	ΔAIC_qh_
	None	Low	High							
Sex	0.113 ♀ > ♂	−0.304 ♀ < ♂	0.191 ♀ > ♂	2	5.225	0.073	113.299	1.23	2.920	−0.177
Heterozygosity	0.687 ↑ as heterozygosity increases	0.349 ↑ as heterozygosity increases	−1.036 ↓ as heterozygosity increases	2	4.704	0.095	112.777	0.70	2.910	−0.187
Age pup	−0.383 pups < yearlings	0.106 pups > yearlings	0.277 pups > yearlings	4	8.364	0.079	112.44	0.37	2.715	−0.382
Age adult	−0.215 adults < yearlings	−0.018 adults < yearlings	0.234 adults > yearlings							
Geographic origin	−0.302 immigrants < natives	−0.124 immigrants < natives	0.426 immigrants > natives	2	8.989	0.011	117.063	4.99	2.994	−0.103
Population size	−0.010 ↓ as population size increases	0.013 ↑ as population size increases	−0.002 ↓ as population size increases	2	2.322	0.31	110.395	−1.68	2.863	−0.234

Tests for significance of each parameter used log-likelihood ratio tests (G). Values for the Akaike Information Criterion (AIC) and the quasi-likelihood information criterion (AICqh) are shown for each alternative model when the specific predictor was removed. For the full model, AIC was 112.074 and AICqh was 3.097.

*Global change of the probability of each of the three levels of *Taenia* abundance in response to a change in the value of each predictor variable. The sum of the values for each predictor is 0, as an increase in the probability in one level must be compensated for by a decrease in other levels.

**Table 2:** Cysticercoses prevalence in ungulates recovered from wolf territories (sample sizes n_wt_) and the control area (sample sizes n_ca_).

Intermediate host	Sample sizes		Cestode species	Number of cases (%)		Fisher’s exact test
	n_wt_	n_ca_		Wolf territories	Control area	p value
Fallow deer (*D. dama*)	7	28	*T. krabbei*	0 (0.0%)	0 (0.0%)	1.0
			*T. hydatigena*	0 (0.0%)	0 (0.0%)	1.0
Red deer (*C. elaphus*)	82	20	*T. krabbei*	3 (3.7%)	1 (5.0%)	1.0
			*T. hydatigena*	5 (6.1%)	0 (0.0%)	0.58
Roe deer (*C. capreolus*)	105	72	*T. krabbei*	5 (4.8%)	1 (1.4%)	0.40
			*T. hydatigena*	2 (1.9%)	1 (1.4%)	1.0
Wild boar (*S. scrofa*)	88	38	*T. krabbei*	0 (0.0%)	0 (0.0%)	1.0
			*T. hydatigena*	5 (5.7%)	0 (0.0%)	0.32

**Table 3:** Response (A–E) and predictor variables used in statistical models regarding wolves (F–J) and ungulates (K–M).

Variable in statistic model	Explanation	Units
A) helminth species richness	count of genetically confirmed helminth species per wolf	continuous data [number of species]
B) helminth diversity	diversity of helminths per wolf 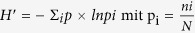	continuous data [Shannon index]
C) *Taenia* spp. abundance category	estimate of *Taenia* spp. abundance documented during dissection per wolf	categorical data (‘none’, ‘low’, ‘high’ abundance)
D) *Sarcocystis* species richness	count of genetically confirmed *Sarcocystis* species per wolf	continuous data [number of species]
E) metacestode infection status	presence of *Taenia* spp. cysts in ungulates	categorical data (‘infected’, ‘not infected’)
F) age	wolf age classed in ecologically relevant and commonly used categories: 0–12 months: ‘pup’; >12 months – 24 months: ‘yearling’; >24 months: ‘adult’^110^	categorical data (‘pup’, ‘yearling’, ‘adult’)
G) heterozygosity	individual heterozygosity as proportion of heterozygous loci (nH) and analysed loci (nL) of microsatellite (H_indiv_ = nH/nL)	continuous data (0–1) [-]
H) geographic origin	genetic affiliation to a known German pack (‘native’) or unknown pack (‘immigrant’)	categorical data (‘immigrant’, ‘native’)
I) population size	annually recorded number of reproducing wolf packs in Germany	continuous data [number of packs]
J) sex	wolf sex determined by dissection	categorical data (‘male’, ‘female’)
K) ungulate species	ungulate species known to be preyed on by wolves in Germany	categorical data (‘roe deer’, ‘red deer’, ‘fallow deer’, ‘wild boar’)
L) study area	ungulate sample collection sites depending on permanent wolf presence or absence	categorical data (‘present’, ‘absent’)
M) metacestode species	Taenia species determined by PCR and sequencing isolated from ungulates	categorical data (‘*T. krabbei*’, ‘*T. hydatigena*’)
