# Supplementary material for: Population expansion and individual age affect endoparasite richness and diversity in a recolonising large carnivore population
Source: Sci Rep. 2017 Jan 27;7:41730. doi: 10.1038/srep41730 (PMC5269671; doi:10.1038/srep41730)
Supplement: Supplementary Material [file srep41730-s1.pdf]

Supplementary Information

Population expansion and individual age affect endoparasite richness and diversity in a recolonising large carnivore population

Ines Lesniak [a,*], Ilja Heckmann [a], Emanuel Heitlinger [a,d], Claudia A. Szentiks [a], Carsten Nowak [b], Verena Harms [b], Anne Jarausch [b], Ilka Reinhardt [c], Gesa Kluth [c], Heribert Hofer [a], Oliver Krone [a]

[a] Leibniz Institute for Zoo and Wildlife Research, Alfred-Kowalke-Straße 17, 10315 Berlin, Germany
[b] Senckenberg Research Institute and Natural History Museum Frankfurt, Clamecystrasse 12, 63571 Gelnhausen, Germany
[c] LUPUS Institute for Wolf Monitoring and Research in Germany, Dorfstraße 20, 02979 Spreewitz, Germany
[d] Humboldt-Universität zu Berlin, Ecology and Evolution of Molecular Parasite Host Interactions, Philippstraße 13, 10115 Berlin, Germany

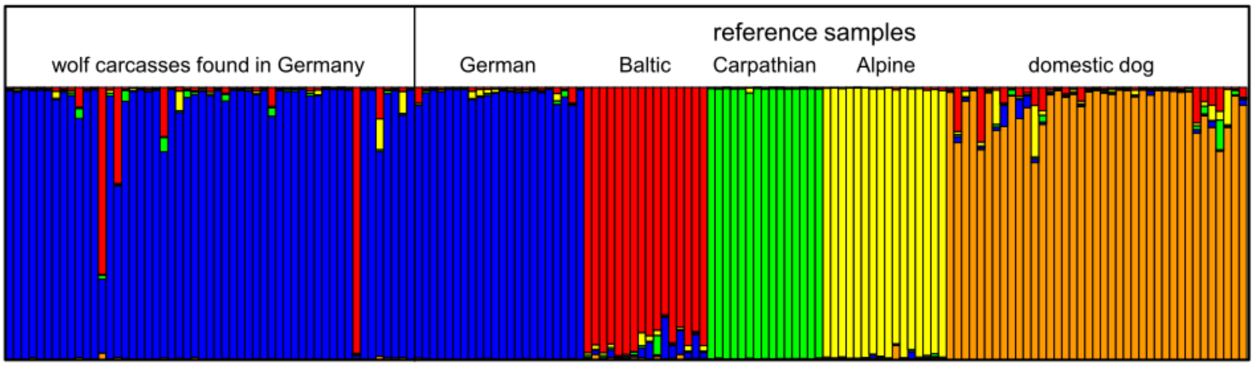

Supplementary Figure S1: Bayesian clustering of 53 wolf carcasses found in Germany in comparison to reference wolf genotypes from Germany (blue), the Baltic region (red), the Carpathians (green), the Alpine region (yellow), and domestic dogs (orange). Each individual is represented by a vertical line divided into five coloured segments that represent the individual's estimated membership fractions in each cluster (K=5).

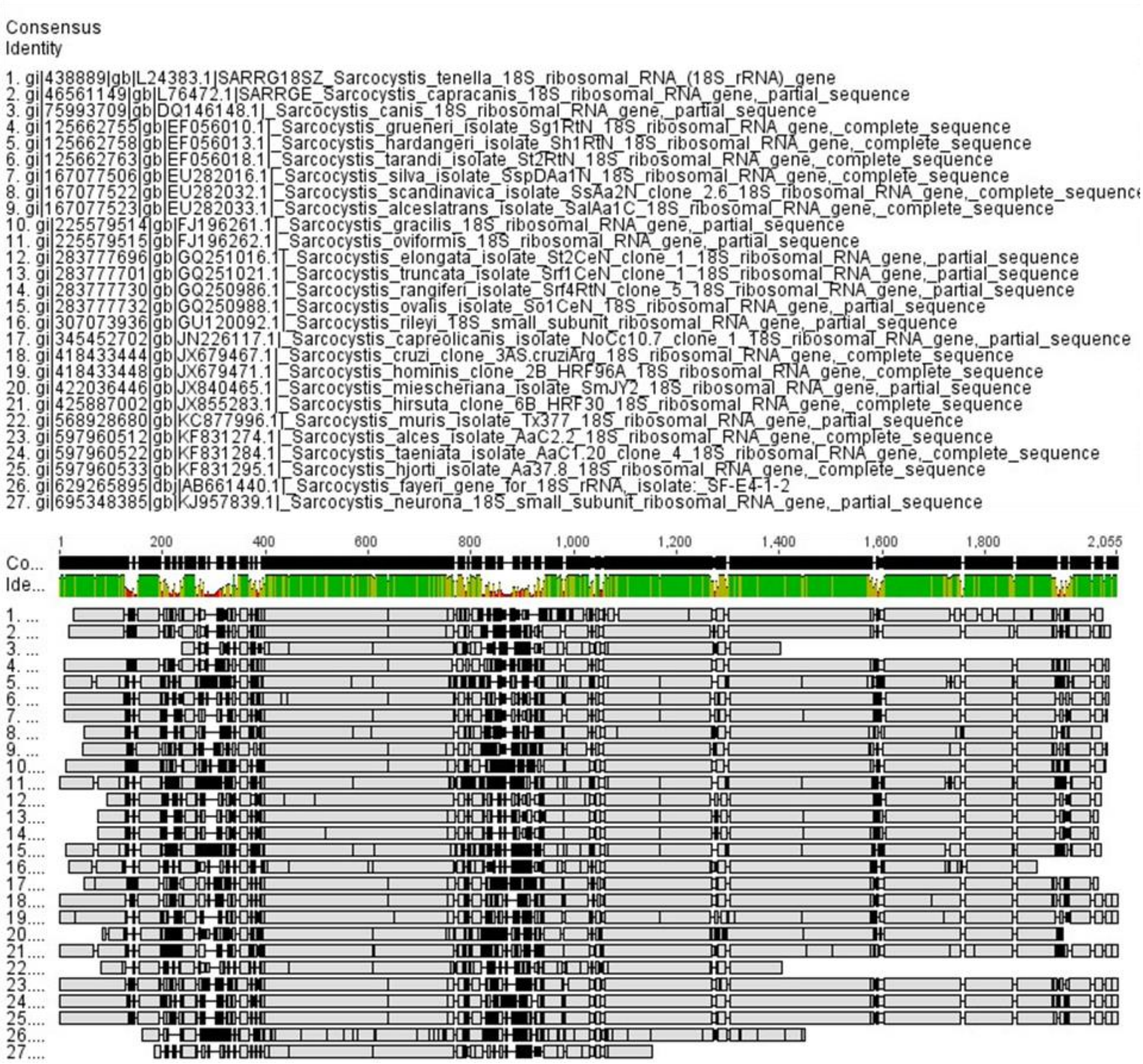

Supplementary Figure S2: The *Sarcocystis* spp. 18S rRNA consensus sequence used for the amplicon design in this study is based on the alignment of 27 different mammalian *Sarcocystis* species.

Supplementary Table S1 Genetic identification of wolf helminths was performed for a subset of parasites. Helminth species were determined based on cox1 and 18S rRNA sequences which were searched in the NCBI nucleotide database. If there was no distinct best hit species, the search was either repeated using an alternative marker gene or morphological indicators were used to determine the species. *Trichinella* spp. were identified by the National Reference Laboratory for *Trichinella* and are not included in this table.

This table is provided as separate online csv file

Supplementary Table S2 Target-specific primers for the amplification of the variable regions of the *Sarcocystis* spp. 18S rRNA gene. *designed for this study

#	primer name	sequence (5'-3')	source	primer position	~product size [bp]
1	G18S4	GCTTGTCTCAAAGATTAAGCCN	1	forward	377
	Euk360_CR	TCTCAKGCKCCYTCTCCG	2	reverse	
2	G18S4	GCTTGTCTCAAAGATTAAGCCN	1	forward	437
	SSU22R	GCCTGCTGCCTTCCTTGG	1	reverse	
3	proti15	TGCCAGTAGTCATATGCTTGTYT	*	forward	378
	proti440_R	CAGGCYCSCTCTCCGGA	*	reverse	
4	proti15	TGCCAGTAGTCATATGCTTGTYT	*	forward	421
	proti482_R	KTTSCGCGCCTGCTGCC	*	reverse	
5	proti15	TGCCAGTAGTCATATGCTTGTYT	*	forward	572
	proti643_R	GAGCTGGAATTACCGCGG	*	reverse	
6	proti89	CKGCGVATGGCTCATTAMAWC	*	forward	485
	proti628_R	GGSTGCTGGCACCAGAC	*	reverse	
7	proti89	CKGCGVATGGCTCATTAMAWC	*	forward	501
	proti643_R	GAGCTGGAATTACCGCGG	*	reverse	
8	F-556	CAGCAGCCGCGGTAATTCC	3	forward	383
	Nem_0425_4R	ARACATTCTTGGCAAATGCYTTC	*	reverse	
9	18S rRNA#1	TGGTGCCAGCAGCCGC	4	forward	577
	Mach2Gen	TCCGTCAATTTCTTTAAGTTTCAG	*	reverse	
10	18S rRNA#1	TGGTGCCAGCAGCCGC	4	forward	389
	Nem_0425_4R	ARACATTCTTGGCAAATGCYTTC	*	reverse	
11	18S rRNA#1	TGGTGCCAGCAGCCGC	4	forward	391
	Nem_0425_2	GAARACATTCTTGGCAAATGCY	*	reverse	
12	18S r DNA549-566	GAGGGCAAGTCTGGTGCC	5	forward	637
	R-1200	CCCGTGTTGAGTCAAATTAAGC	3	reverse	
13	proti628	GTCTGGTGCCAGCASCC	*	forward	604
	18S rDNA 1141-1161	GGTGCCCTTCCGTCAATTC	5	reverse	
14	18S r RNA549-566	GAGGGCAAGTCTGGTGCC	5	forward	588
	Mach2Gen	TCCGTCAATTTCTTTAAGTTTCAG	*	reverse	
15	18S r DNA573-589	GCCGCGGTAATTCCAGCT	5	forward	378
	Nem_0425_4R	ARACATTCTTGGCAAATGCYTTC	*	reverse	

Supplementary Table S3 Comparison of helminth species richness (HSR) and helminth prevalence (%) between wolves from the CEL wolf population (this study) and the Baltic wolf population. Red p-values indicate significant differences.

	this study	Shimalov *et al.*[6]			Bagrade *et al.*[7]			Szczęsna-Staśkiewicz[8]			Moks *et al.*[9]		
	**Germany**	**Belarus**	**p-value**	**t**	**Latvia**	**p-value**	**t**	**Poland**	**p-value**	**t**	**Estonia**	**p-value**	**t**
n wolves	53	52			34			18			26		
HSR	13	24			17			17			13		
mean $HSR_{individual}$	2.57±0.26	NA			4.6±0.35	**0.002**	-3.422	4.6±0.54	**$2.024e^{-5}$**	-4.5796	NA		
max. $HSR_{individual}$	8	NA			8			9			NA		
Taenia spp richness	2	5			7			5			5		
	**%**	**%**	**p-value**	X^2	**%**	**p-value**	X^2	**%**	**p-value**	X^2	**%**	**p-value**	X^2
C. plica	25	14	0.232	1.429	41	0.161	1.961	NA	NA	NA	0	**0.015**	5.954
C. vulpis	25	8	**0.038**	4.312	9	0.118	2.438	17	0.716	0.132	0	**0.015**	5.954
C. aerophila	15	0	**0.011**	6.487	36	0.054	3.701	17	1.000	$3137e^{-30}$	NA	NA	NA
U. stenocephala	11	15	0.745	0.106	41	**0.003**	8.810	72	**$2.196e^{-6}$**	22.415	77	**$2.46e^{-8}$**	31.093
T. canis	11	21	0.270	1.126	6	0.634	0.227	11	1.000	$5.480e^{-30}$	8	0.916	0.011
T. leonina	4	14	0.154	2.029	0	0.680	0.170	6	1.000	$5.304e^{-30}$	8	0.841	0.040
Trichinella spp.	4	19	0.052	3.785	70	**$5.103e^{-6}$**	20.798	33	0.371	0.800	50	**$3.887e^{-6}$**	21.320
T. krabbei	77	8	**$2.298e^{-12}$**	49.212	9	**$1.756e^{-9}$**	36.227	NA	NA	NA	15	**$6.171e^{-7}$**	24.858
T. hydatigena	15	27	0.212	1.561	41	**0.013**	6.141	56	**0.002**	9.584	12	0.934	0.007
M. litteratus	9	0	0.070	3.281	0	0.170	1.884	NA	NA	NA	0	0.260	1.269
E. multilocularis	2	0	1.000	$1.262e^{-29}$	6	0.693	0.156	0	1.000	$4.819e^{-29}$	0	1.000	$1.478e^{-29}$
A. alata	53	17	**0.001**	12.998	85	**0.004**	8.279	33	0.247	1.340	89	**0.004**	8.818
total	89	80	0.392	0.733	100	0.110	2.559	100	0.317	1.003	100	0.183	1.777

Supplementary Table S4 Comparison of cysticercoses prevalence (%) between ungulates from Germany and Eastern Europe. Red p-values indicate significant differences.

**host species**		this study **Germany**	Murai *et al.*[10] **Hungary**			Kuzmina *et al.*[11] **Ukraine**			Onac *et al.*[12] **Romania**		
n roe deer (*Capreolus capreolus*)		177	345			92			NA		
n red deer (*Cervus elaphus*)		102	70			NA			21		
n wild boar (*Sus scrofa*)		126	44			NA			267		
n fallow deer (*Dama dama*)		35	35			NA			NA		
	**cestode species**	**%**	**%**	**p-value**	X^2	**%**	**p-value**	X^2	**%**	**p-value**	X^2
roe deer	*T. krabbei*	0	33	**$8.64e^{-14}$**	55.654	0	0.177	1.825	NA	NA	NA
	T. hydatigena	2	18	**$2.89e^{-07}$**	26.325	2	1	$1.64e^{-29}$	NA	NA	NA
red deer	*T. krabbei*	4	19	**0.004**	8.4254	NA	NA	NA	NA	NA	NA
	T. hydatigena	5	36	**$4.98e^{-07}$**	25.271	NA	NA	NA	NA	NA	NA
	E. granulosus	0	0	NA	NA	NA	NA	NA	12	**0.0282**	4.818
wild boar	*T. hydatigena*	4	43	**$6.42e^{-10}$**	38.189	NA	NA	NA	NA	NA	NA
	E. granulosus	0	36	**$9.63e^{-12}$**	46.403	NA	NA	NA	10	**$8.55e^{-05}$**	15.432
fallow deer	*T. krabbei*	0	6	0.4731	0.51471	NA	NA	NA	NA	NA	NA
	T. hydatigena	0	20	**0.017**	5.7143	NA	NA	NA	NA	NA	NA

References

1 Dorris, M., Viney, M. E. & Blaxter, M. L. Molecular phylogenetic analysis of the genus Strongyloides and related nematodes. *Int J Parasitol* **32**, 1507-1517 (2002).

2 Behnke, A. *et al.* Microeukaryote community patterns along an O2/H2S gradient in a supersulfidic anoxic fjord (Framvaren, Norway). *Applied and environmental microbiology* **72**, 3626-3636, doi:10.1128/AEM.72.5.3626-3636.2006 (2006).

3 Hadziavdic, K. *et al.* Characterization of the 18S rRNA gene for designing universal eukaryote specific primers. *PloS one* **9**, e87624, doi:10.1371/journal.pone.0087624 (2014).

4 Machida, R. J. & Knowlton, N. PCR primers for metazoan nuclear 18S and 28S ribosomal DNA sequences. *PloS one* **7**, e46180, doi:10.1371/journal.pone.0046180 (2012).

5 Wang, Y., Tian, R. M., Gao, Z. M., Bougouffa, S. & Qian, P.-Y. Optimal Eukaryotic 18S and Universal 16S/18S Ribosomal RNA Primers and Their Application in a Study of Symbiosis. *PloS one* **9**, e90053, doi:10.1371/journal.pone.0090053 (2014).

6 Shimalov, V. V. & Shimalov, V. T. Helminth fauna of the wolf (Canis lupus Linnaeus, 1758) in Belorussian Polesie. *Parasitology research* **86**, 163-164 (2000).

7 Bagrade, G., Kirjusina, M., Vismanis, K. & Ozolins, J. Helminth parasites of the wolf Canis lupus from Latvia. *Journal of helminthology* **83**, 63-68, doi:10.1017/S0022149X08123860 (2009).

8 Szczęsna-Staśkiewicz, J. *Helmintofauna wilka Canis lupus L. w Polsce.* , Wrocław University of Environmental and Life Sciences, (2009).

9 Moks, E. *et al.* Helminthologic survey of the wolf (Canis lupus) in Estonia, with an emphasis on Echinococcus granulosus. *Journal of wildlife diseases* **42**, 359-365, doi:10.7589/0090-3558-42.2.359 (2006).

10 Murai, É., Sugár, L. . Taeniid species in Hungary (Cestoda, Taenidae). I. Cysticercosis, coenurosis and hydatidosis of wild ungulates *Parasitologia Hungarica* **12**, 41-52 (1979).

11 Kuzmina, T., Kharchenko, V., Malega, A. Helminth Fauna of Roe Deer (Capreolus Capreolus) in Ukraine: Biodiversity and Parasite Community. *Vestnik Zoologii* **44**, 12-19, doi: 10.2478/v10058-010-0002-1 (2010).

12 Onac, D., Győrke, A., Oltean, M., Gavrea, R. & Cozma, V. First detection of Echinococcus granulosus G1 and G7 in wild boars (Sus scrofa) and red deer (Cervus elaphus) in Romania using PCR and PCR-RFLP techniques. *Vet Parasitol* **193**, 289-291, doi:http://dx.doi.org/10.1016/j.vetpar.2012.11.044 (2013).
